# Effect of air flow on tubular solar still efficiency

**DOI:** 10.1186/1735-2746-10-31

**Published:** 2013-04-15

**Authors:** Arunkumar Thirugnanasambantham, Jayaprakash Rajan, Amimul Ahsan, Vinothkumar Kandasamy

**Affiliations:** 1 Department of Physics, Dr. NGP Institute of Technology, Coimbatore 641 048 Tamilnadu, India; 2 Department of Physics, Sri Ramakrishna Mission Vidyalaya College of Arts and Science, Coimbatore 41 020 Tamilnadu, India; 3 Research and Development (Renewable Energy), NSP Green Energy Technologies Pvt. Ltd. Chennai, Tamilnadu, India

**Keywords:** Compound Parabolic Concentrator, Desalination, Tubular Solar Still

## Abstract

**Background:**

An experimental work was reported to estimate the increase in distillate yield for a compound parabolic concentrator-concentric tubular solar still (CPC-CTSS). The CPC dramatically increases the heating of the saline water. A novel idea was proposed to study the characteristic features of CPC for desalination to produce a large quantity of distillate yield. A rectangular basin of dimension 2 m × 0.025 m × 0.02 m was fabricated of copper and was placed at the focus of the CPC. This basin is covered by two cylindrical glass tubes of length 2 m with two different diameters of 0.02 m and 0.03 m. The experimental study was operated with two modes: without and with air flow between inner and outer tubes. The rate of air flow was fixed throughout the experiment at 4.5 m/s. On the basis of performance results, the water collection rate was 1445 ml/day without air flow and 2020 ml/day with air flow and the efficiencies were 16.2% and 18.9%, respectively.

**Findings:**

The experimental study was operated with two modes: without and with air flow between inner and outer tubes. The rate of air flow was fixed throughout the experiment at 4.5 m/s.

**Conclusions:**

On the basis of performance results, the water collection rate was 1445 ml/day without air flow and 2020 ml/day with air flow and the efficiencies were 16.2% and 18.9%, respectively.

## Introduction

Ahsan and Fukuhara [1] studied on a new heat and mass transfer tubular solar still and found that the heat and mass transfer coefficients can be expressed as functions of the temperature difference between the saline water and the cover. Ahsan *et al*. [2], experimentally studied on the evaporation, condensation and production of a tubular solar still and found that the relative humidity of the humid air was definitely not saturated and the hourly evaporation, condensation and production fluxes were proportional to the humid air temperature and relative humidity. Ahsan and Fukuhara [3-5] analyzed on a tubular solar still and on a quasi steady heat and mass transfer due to evaporation and condensation taking an account of humid air properties inside the still and found that the analytical solutions derived from the present model could reproduce the experimental results perfectly. Arunkumar *et al*. [6], studied on a hemispherical solar still.

Numerous research activities have been done with CPCs such as cost-effective asymmetric CPCs [7], non-modified absorbers [8], solar powered adsorption refrigerator with CPC collection system [9], non-imaging solar collector [10], non-tracking solar concentrators [11], and non-evacuated solar collectors [12].

Singh *et al*. [13], studied on the design parameters for concentrator assisted solar distillation system. Analytical expressions for the water and glass cover temperatures, the rate of heat flux due to evaporation, the rate of distillate output and the instantaneous thermal efficiency have been derived in terms of the system design and climatic parameters. It is analytically shown that the efficiency of the system with a concentrator was higher than that with a collector.

Chaochi *et al*. [14], designed and built a small solar desalination unit equipped with a parabolic concentrator and observed that the maximum efficiency corresponds to the maximum solar lighting obtained towards 14:00. At that hour, the boiler was nearly in a horizontal position, which maximizes the offered heat transfer surface. Mohamad and El-Minshawy [15] dealed with the status of solar energy as clean and renewable energy applications in desalination.

A novel idea proposed in this work is the CPC’s absorber which is acting as a saline water basin in a tubular arrangement. This specially designed CPC-CTSS required only 3 minutes warming up, as opposed to a typical warm-up period of an hour or more for basin type stills with no reflectors. The inner tube was cooled by flowing air at the rate of 4.5 m/s.

### Design of the CPC

**Figure 1:**
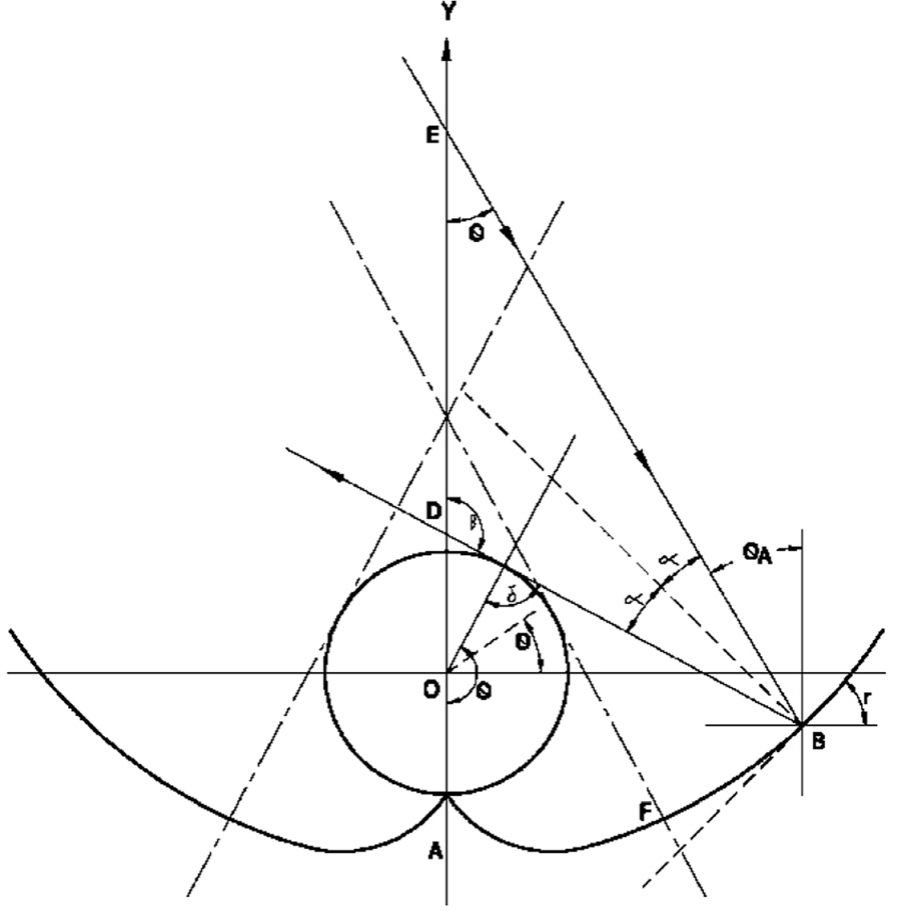
Reflector shape for tubular absorber.

The reflector profile for the CPC [16] with a ‘V’ groove at the bottom is such that all rays entering the cavity opening end up at the absorber. It can be drawn for a tubular absorber of radius r_1_, and half acceptance angle θ_A_ allowing a small space δ between cavity opening and the absorber see Figure 1.

(1) $$x=r_{1}\sin\theta -\rho \cos\theta$$

The upper portion of the reflector profile AB and CD can be generated using the relation,

(2) $$x=-r_{1}\cos\theta -\rho \sin\theta$$

Where;

ρ is the ground reflectance:

(3) $$\rho =r_{1}\left[\theta +\Delta \theta \right]$$

For the angles of

$$\pi -\left(2\varphi +\epsilon \right)\leq \theta \leq \theta_{A}+\pi /2$$

(4) $$\rho =\frac{r_{1}\left[\theta +\theta_{A}+\pi /2+2\Delta \theta -\cos\left(\theta -\theta_{A}\right)\right]}{\left[1+\sin\left(\theta -\theta_{A}\right)\right]}$$

For the range of angles:

$$\pi /2+\theta_{A}\leq \theta \leq 3\pi /2-\theta_{A}$$

Where;

(5) $$\Delta \theta =\cot\varphi -\left[\pi -\left(2\varphi +\epsilon \right)\right]$$

And, from the Figure 1:

(6) $$\varphi =\sin^{-1}\left(r_{1}/r_{2}\right)$$

(7) $$\varphi +\epsilon =\sin^{-1}\left[\left(r_{1}+\delta \right)/r_{2}\right]$$

The upper portion of the radius of envelope is:

(8) $$r_{2}=\frac{{\left[r_{1}{}^{2}+{\left(\delta /2\sin\alpha \right)}^{2}+\delta r_{1}\right]}^{1/2}}{\cos\alpha }$$

(9) $$\left(1/\sin\alpha \right)-\sin\alpha =\delta /2r_{1}$$

The bottom region of the profile can be modified by incorporating a V-shaped reflector portion just below the absorber.

The height ‘h’ and the open angle ‘2Ψ’ of the ‘V’ groove are related by the following relations:

(10) π−2α≤2ψ≤π/2+α

(11) $$\sin\alpha =r_{1}/\left(r+\delta +h\right)$$

Average fraction of radiation lost ‘L’ is given by:

(12) $$L\approx \frac{\epsilon^{2}}{2\left(1+2\varphi \tan\varphi \right)}$$

And the ratio of the concentration achieved to the maximum possible concentration of the concentrator is calculated as:

(13) $$\frac{\mathrm{CR}}{CR_{\max}}=\frac{\cot\phi +2\phi +\epsilon }{\pi }$$

Where;

(14) $$CR_{\max}=1/\sin\theta_{A}$$

## Materials and methods

A 2 m concentric tubular solar still was designed and fabricated. The specification of the tubular solar still is shown in Table 1. The inner and outer tubes were positioned with a 5 mm gap for the flowing air to cool the outer surface of the inner tube. An air blower was used to propel the air inside the tube at constant rate of 4.5 m/s. A rectangular basin of dimension 2 m × 0.03 m × 0.025 m was designed and coated with black paint using a spray technique. The surface was free of dust, dirt, rust and moisture before spraying. During operation, the water level in the basin decreased due to fast evaporation from the basin, so a dry spot appeared in the basin. This was avoided in successive trials by flowing the water continuously in the still with the help of a graduated tube. This continuous supply of water was maintained by a water storage tank which was kept near to the CPC still. The outlet of the storage tank was connected to the inlet of the CPC still. After saline water was poured inside the basin, the entire arrangement was sealed properly by using a rubber cork to ensure no air leakage. The cooling air had a constant flow rate of 4.5 m/s. Microprocessor digital anemometer (AM 4201) with anemometer vane probe was used to measure the flow rate of air inside the concentric tubes. The flow meter was calibrated for the actual flow rate at the operating temperature range. The process was repeated many times to assure a good accuracy and each time the flow rate were measured for a 1 minute interval.

**Table 1:** Still technical and operation details

**S. no**	**Climatic conditions**	**Parameter**	**Value**
1	Clear sky	Solar radiation (W/m^2^)	652 - 1159
		Ambient temperature (°C)	26.2 - 34
		Relative humidity (%)	55 - 21
		Average wind velocity (m/s)	1
2	Design	Basin absorption (α_b_)	0.96
		Absorbtivity of cover (α_g_)	0.05
		Reflectance of cover	0.05
		Transmittance of cover (τ_g_)	0.9
		Specific heat of water (C_w_)	4190 J/(kg.K)
		Length (m)	2
		Width (m)	0.102
		Radius of the receiver (m)	0.0158
		Radius of the envelope (m)	2.2 x 10^-2^ m

**Figure 2:**
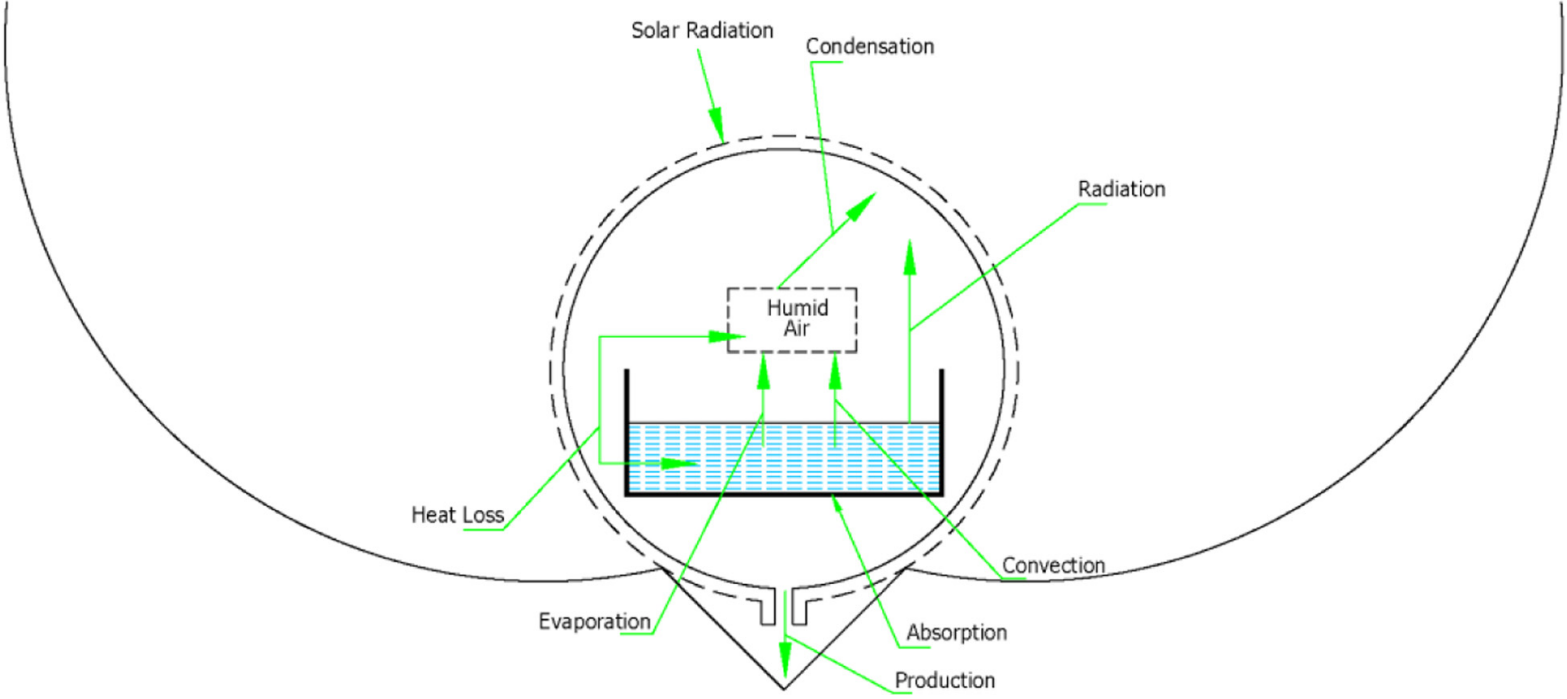
Cross sectional view of CPC-CTSS.

**Figure 3:**
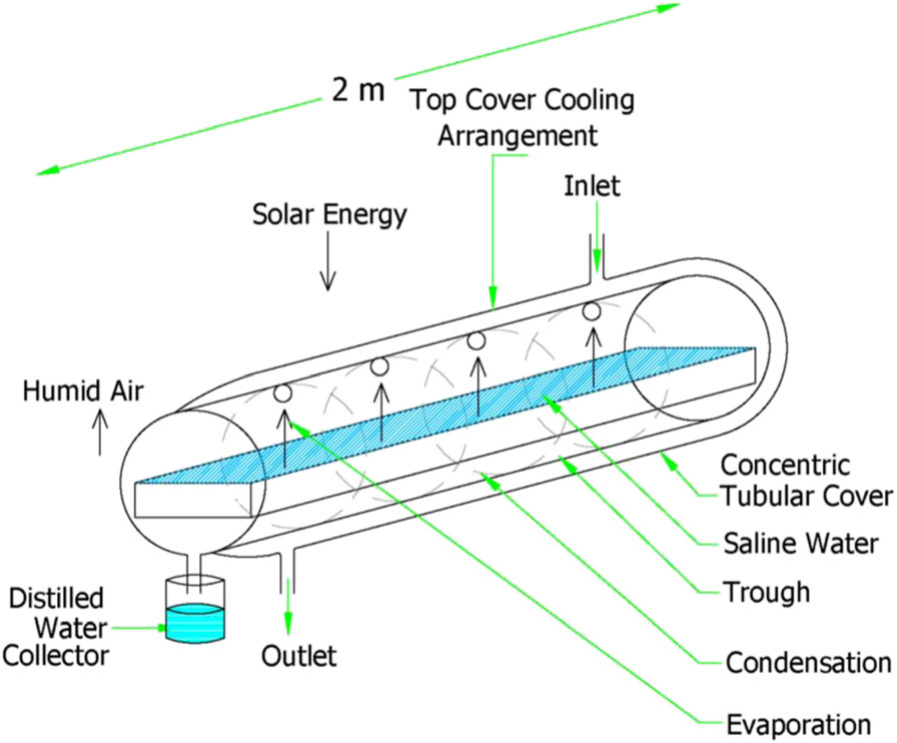
Schematic view of CPC-CTSS.

**Figure 4:**
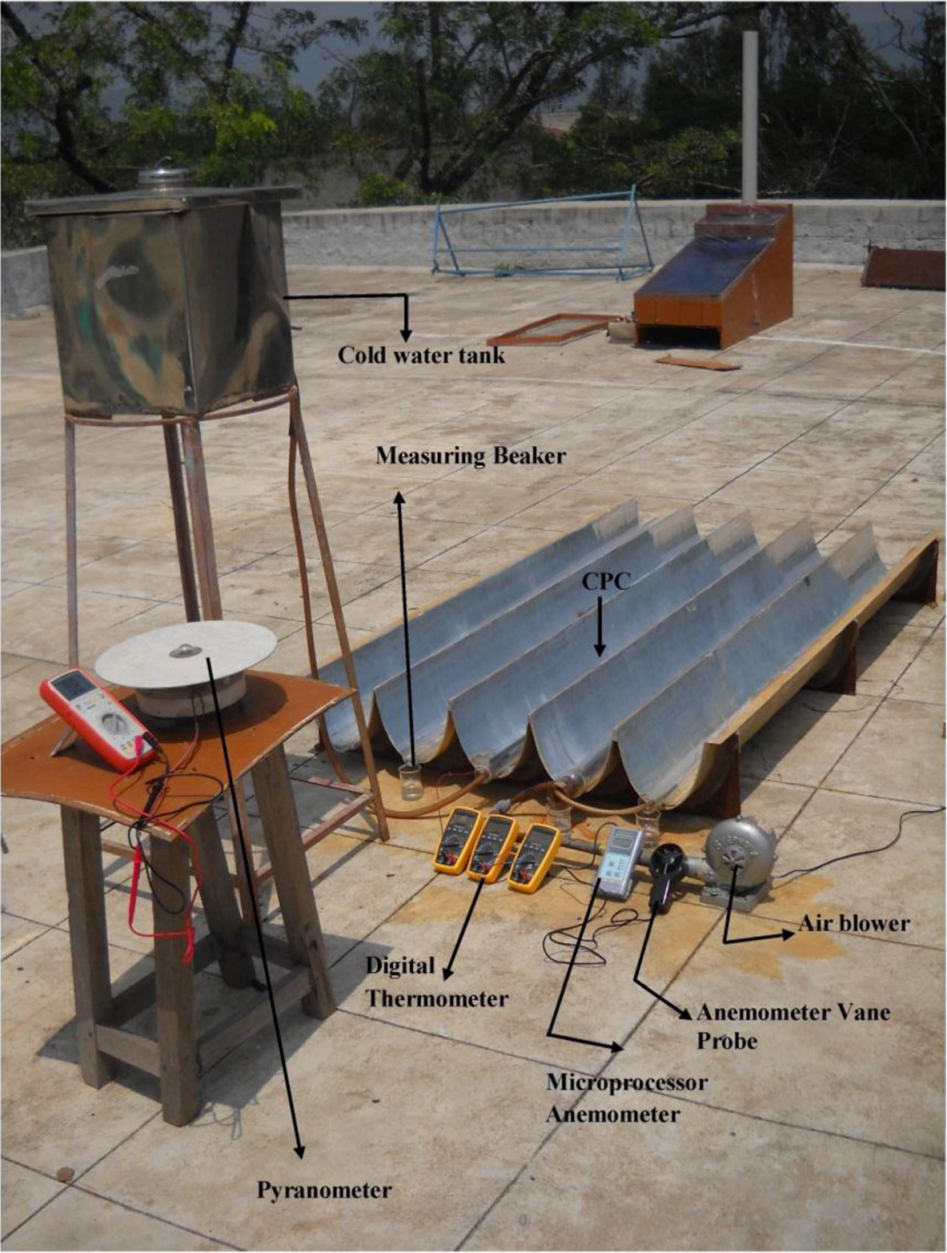
Pictorial view of air blower arrangement.

The following parameters were measured every ten minutes: water temperature (T_w_), interior humid air temperature (T_a_), ambient temperature (T_amb_), total diffuse solar radiation (I), and distillate yield. The diffuse radiation is measured by a precision Pyranometer. All dimensions of the pictorial and schematic views of the experimental device are shown in Figures 2, 3 and 4.

### Water quality analysis

The water quality analysis is performed at the Tamilnadu Agricultural University’s, Soil Science and Agricultural Chemistry Department in Coimbatore, India. The results obtained are presented in Table 2. Two different water samples (A and B) are tested with their pH and electrical conductivity (in dS/m) measured before and after desalination. Before desalination, the level of electrical conductivity in the water of about 1 dS/m which is ~2% of ocean water, but not drinkable was obtained. However, after desalination it decreased to 0.10 dS/m^-^, which is drinkable. The typical pH varies from one water sample to another as well as on the nature of the construction materials used in the water distribution system. It is usually in the range of 6.5 to 8.

**Table 2:** Tested water quality results

**Sample no.**	**TDS (mg/L)**		**pH**		**Conductivity (Ds/m)**	
	**Before desalination**	**After desalination**	**Before desalination**	**After desalination**	**Before desalination**	**After desalination**
Sample A-B	320	40	7.60	7.32	1	0.10

## Findings

**Figure 5:**
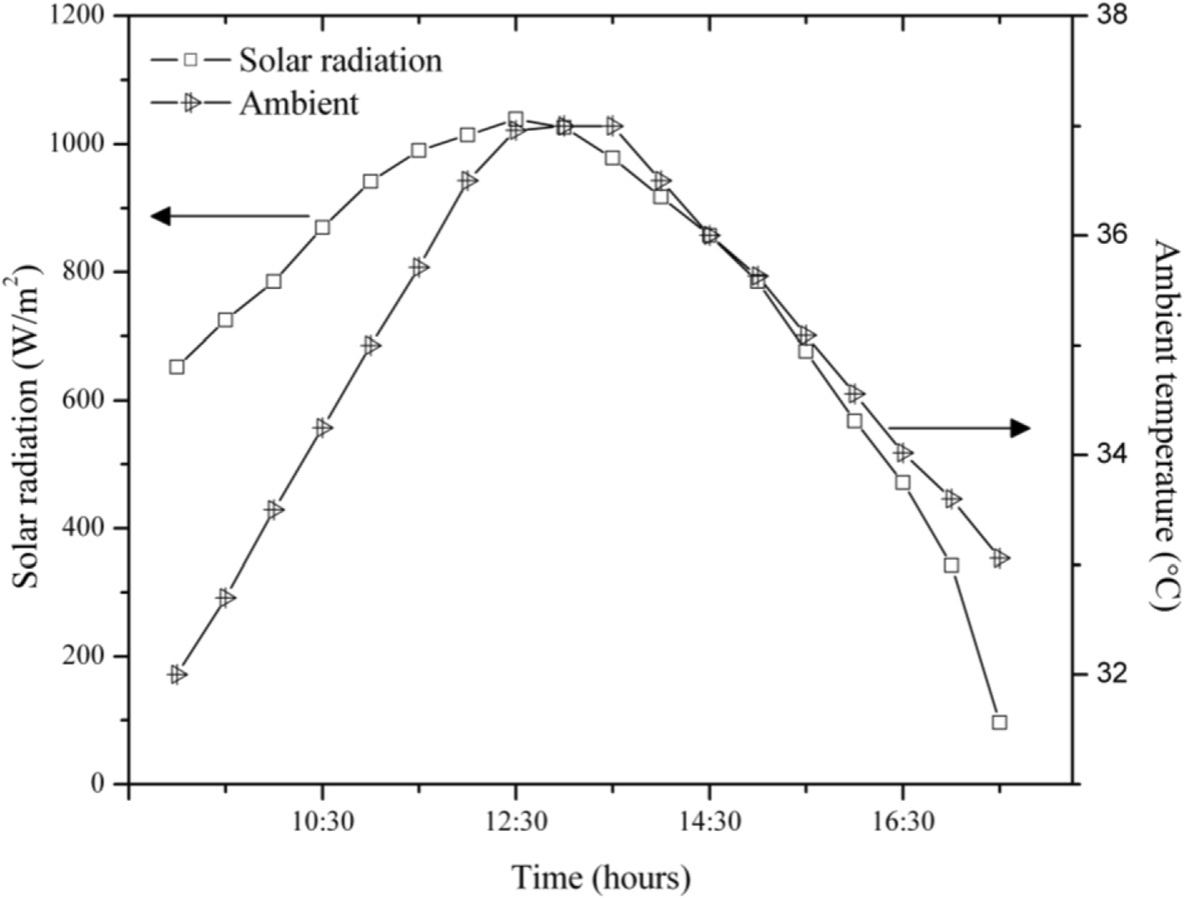
Variation of solar radiation and ambient temperature with respect to time.

**Figure 6:**
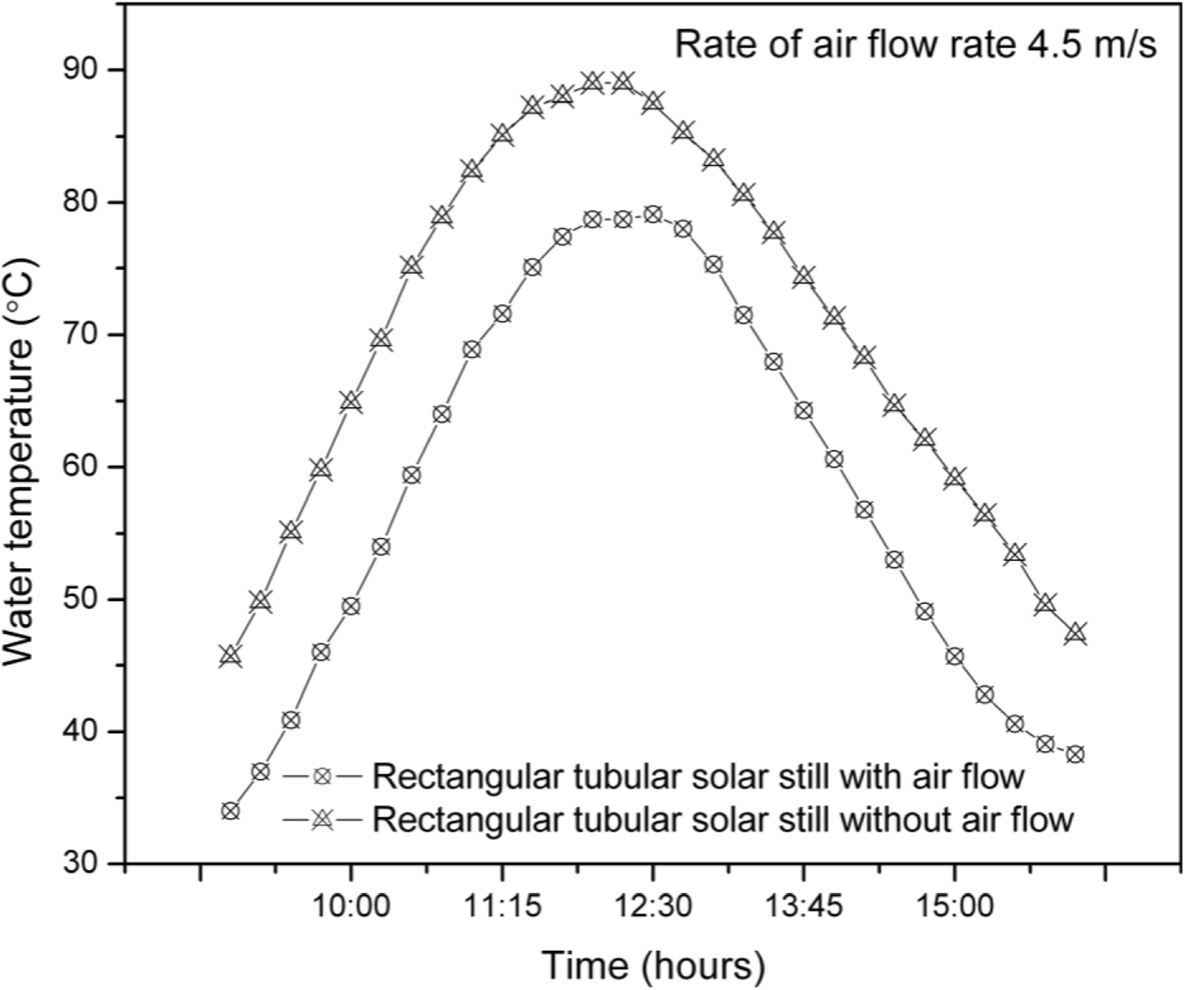
Variation of water temperature with respect to time.

**Figure 7:**
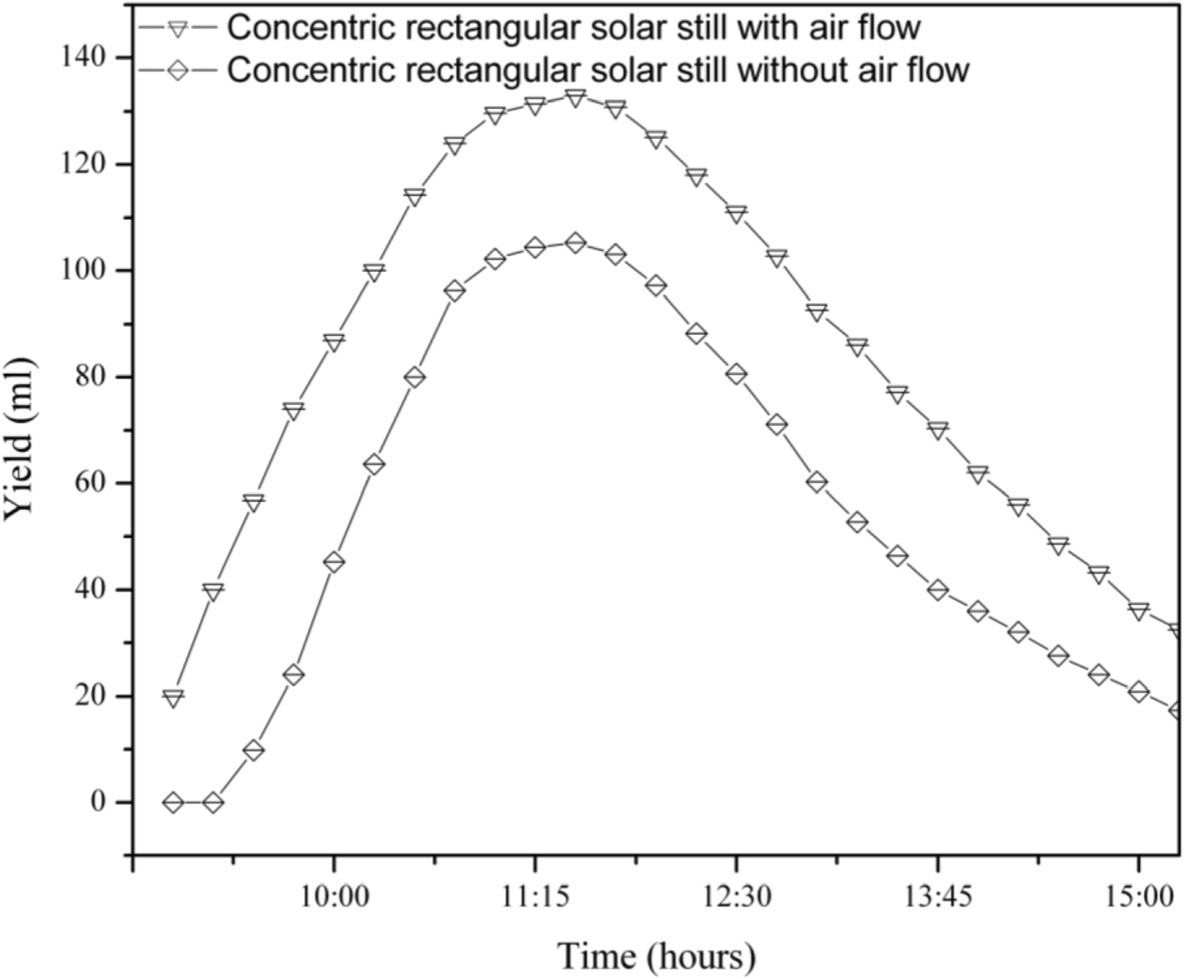
Variation of yield with respect to time.

Ahmed [17] has reported a continuous increase in output from a solar still with the increase in total solar radiation. Hourly variation of daily distillate output depends upon how the radiation is distributed throughout the day. The solar radiation and ambient temperature for a typical day are shown in Figure 5. The variation of ambient temperature was in the range of 24.7°C to 36.2°C and solar radiation received during the study was in the range of 362 W/m^2^ to 1038 W/m^2^. Figure 6 shows the time variation of temperature with air flow over the inner tube. The water temperature was in the range of 45.7°C to 89°C without air flow and 34°C to 79.1°C with air flow. Figure 7 shows the variation of distilled yield with respect to time. The water collection rate obtained was 1428 mL/day without flow of air and 2020 mL/day with air flow. A significant increase in distilled water production was observed in the concentric tubular solar still with air flow over the inner tube. The average daily solar direct and diffuse incident radiation without and with air flow were 7080 Wh/m^2^ and 6960 Wh/m^2^, respectively. The total aperture was 2.04 m^2^. With a heat of vaporization at boiling of 2260 J/g, the overall solar efficiencies without and with air flow were 16.2% and 18.9%, respectively.

## Discussion

**Figure 8:**
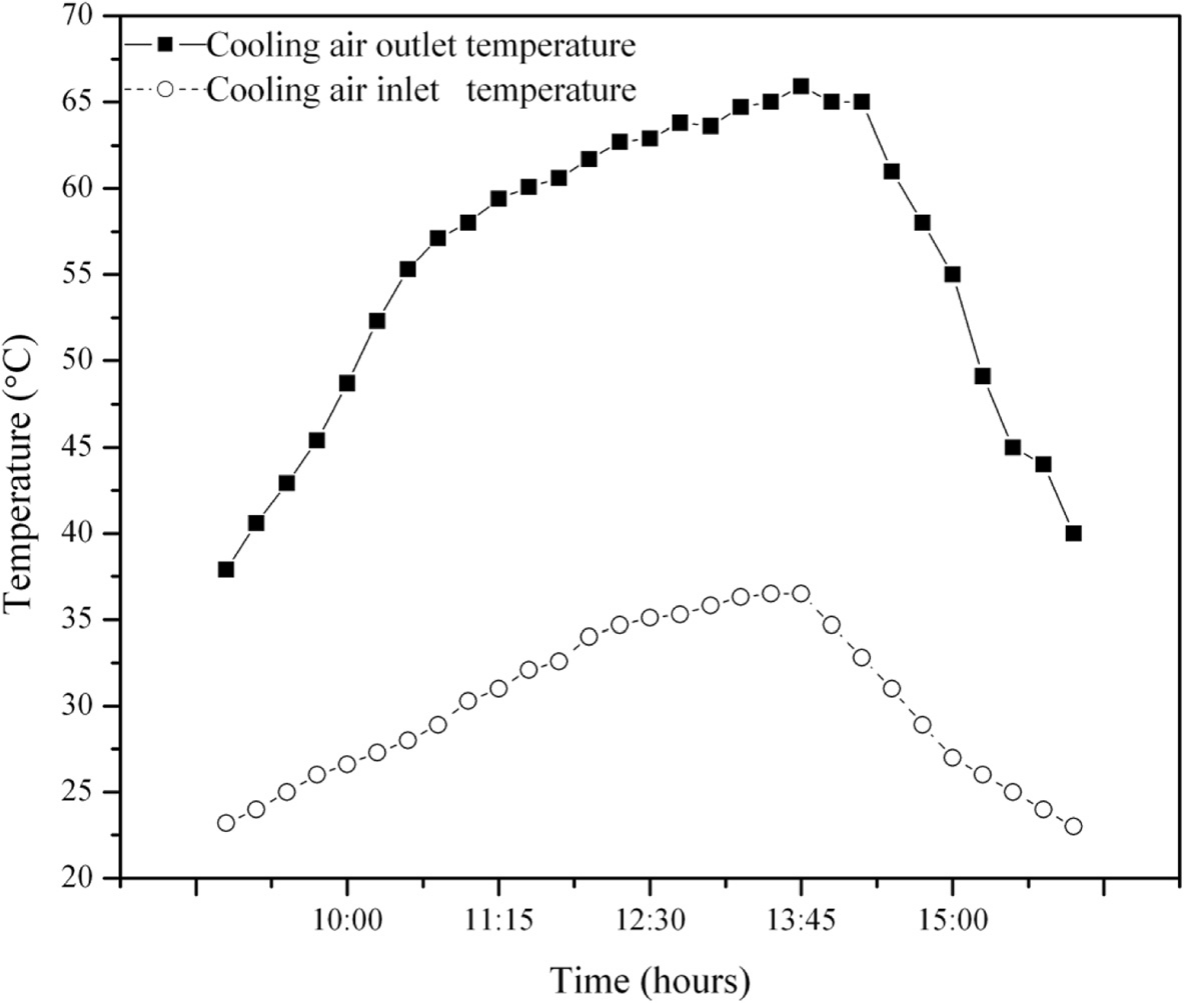
Time variation of output cooling air.

The driving force of the solar distillation technique is the difference between temperature of water in the still basin and the temperature of the cover (Tw-Tc) Arunkumar *et al*., [6]. The existence of such temperature difference ensures the continuation of the distillation process. Clearly, the tubular solar still with top cover air cooling is superior to the solar still without cooling. This increase of output is shown in Figure 7. The productivity of the hemispherical solar still is moderately improved by cooling the top cover. The variation of distilled yield is in the range of 17 ml to 113 ml for without cooling and 32 ml to 132 ml with cooling. The daily average water productivity is increased from 1500 ml to 2200 ml for a fixed flow rate of 4.5 m/s of cold air fed. Figure 8 depicts the diurnal variation of efficiency with respect to time. The overall daily efficiency is calculated with the total radiation and total water output. The conventional still achieved 16.2%, and the cooling air system improved it to 18.9%. This implies that the efficiency of the tubular solar still was improved by a factor of 1.25 due to the effect of air cooling. However, the advantage of the CPC is that the expensive still area is minimized and replaced with relatively inexpensive reflector. Therefore, the overall cost of distilled water should be lower.

The inlet and outlet temperatures [18] of the cooling air are shown in Figure 8. The extracted heat from the CPC-CTSS would allow further production of distilled water in a single slope solar still. With a 25°C rise and 4.5 m/s flow for 8 hours, the amount of heat was 140 Wh. With a 50% efficient single slope still, this could produce an additional 110 ml of distilled water, roughly 5% increase in output and efficiency. Air flow meter was kept near by the system to supply the cooling air. Its temperature was influenced by the ambient temperature and the solar radiation falling on it, which explains the variation in input temperature.

## Conclusion

This research article summarized a new compound parabolic concentric-tubular solar still (CPC-CTSS), which has been designed for and tested under the climatic conditions of Coimbatore, India. The effect of cooling air flowing over the condensation surface was studied. The daily yield of CPC-CTSS was found 1445 mL/day and 16.2% efficiency without air flow and 2020 mL/day and 18.9% efficiency with air flow at a constant flow rate of 4.5 m/s. To bound in a nutshell, this innovative approach of concentrator assisted tubular solar still with air flow augments the performance with enhanced rate of evaporation and condensation with safer operation procedures.

## Competing interests

The authors declare that they have no competing interests.

## Authors’ contributions

Arunkumar Thirugnanasambantham carried out the experiments under the guidance of Jayaprakash Rajan. All authors read and approved the final manuscript.

## References

[B1] Ahsan A Fukuhara T Evaporative mass transfer in tubular solar still Journal of Hydro science and Hydraulic Engineering 2008 26 15 25

[B2] Ahsan A Fukuhara T Condensation mass transfer in unsaturated humid air inside tubular solar still J Hydrosci Hydraul Eng 2010 28 31 42

[B3] Ahsan A Fukuhara T Mass and heat transfer model of tubular solar still Sol Energ 2010 84 1147 1156 10.1016/j.solener.2010.03.019

[B4] Ahsan A Islam KMS Fukuhara T Ghazali AH Experimental study on evaporation, condensation and production of a new tubular solar still Desalination 2010 260 172 179 10.1016/j.desal.2010.04.044

[B5] Ahsan A Imteaz M Rahman A Yusuf B Fukuhara T Design, fabrication and performance analysis of an improved solar still Desalination 2012 292 105 112

[B6] Arunkumar T Jayaprakash R Denkenberger D Ahsan A Okundamiya MS Kumar S Tanaka H Aybar HS An experimental study on a hemispherical solar still Desalination 2012 286 342 348

[B7] Tripanagnostopoulos Y Yianoulis P Papaefthimiou M Souliotis Nousia T Cost effective asymmetric CPC solar collectors Renew Energ 1996 16 628 631

[B8] Khonkar HEI Sayigh AAM Optimization of the tubular absorber using a compound parabolic concentrator Renew Energ 1995 6 17 21 10.1016/0960-1481(94)00061-A

[B9] Manuel IG Luis RR Solar powered absorption refrigerator with CPC collection system: collector design and experimental test Energ Convers Manage 2007 48 2587 2594 10.1016/j.enconman.2007.03.016

[B10] Baum HP Gordon JM Geometric characteristics of ideal non-imaging (CPC solar collectors with cylindrical absorber Sol Energ 1984 33 455 458 10.1016/0038-092X(84)90198-1

[B11] Spirkl W Ries H Muschaweck J Winston R Non tracking solar concentrators Sol Energ 1998 62 113 120 10.1016/S0038-092X(97)00106-0

[B12] Rabl A O’gallagher J Winston R Design and test of non-evacuated solar collectors with compound parabolic concentrators Sol Energ 1980 25 335 351 10.1016/0038-092X(80)90346-1

[B13] Singh SK Bhatnagar VP Tiwari GN Design parameters for concentrator assisted solar distillation system Energ Convers Manage 1996 37 247 252 10.1016/0196-8904(95)00166-B

[B14] Chaochi B Zrelli A Gabsi S Desalination of brackish water by means parabolic solar concentrator Desalination 2007 217 118 126 10.1016/j.desal.2007.02.009

[B15] Mohamad AMI El-Minshawy NA Theoretical investigation of solar humidification-dehumidification system using parabolic trough concentrator Energ Convers Manage 2011 52 3112 3119 10.1016/j.enconman.2011.04.026

[B16] Oommen R Jayaraman S Development and performance analysis of compound parabolic solar concentrators with reduced gap losses: ‘V’ groove reflector Renew Energ 2002 27 259 275 10.1016/S0960-1481(01)00185-9

[B17] Ahmed ST Study of single effect solar still with an internal condenser Int J Solar and Wind Tech 1988 5 637 643 10.1016/0741-983X(88)90061-6

[B18] Rabbani D Hooshyar H Application of flat plate solar collector for thermal disinfection of water effluents Iran J Environ Health Sci Eng 2011 8 117 122

